# What should rheumatologists know about Gaucher disease and Fabry disease? Connecting the dots for an overview

**DOI:** 10.1186/s42358-024-00362-2

**Published:** 2024-03-22

**Authors:** Rafael Alves Cordeiro, Nilton Salles Rosa Neto, Henrique Ayres Mayrink Giardini

**Affiliations:** 1 Division of Rheumatology, Hospital das Clinicas HCFMUSP, Faculdade de Medicina, Universidade de Sao Paulo, Av Dr Arnaldo, 455, 3 andar, sala 3184, Cerqueira Cesar, Sao Paulo, SP CEP 01246-903 Brazil; 2 Centro de Doenças Raras e da Imunidade, Hospital Nove de Julho, São Paulo, Brazil; 3 Universidade Santo Amaro, São Paulo, Brazil

**Keywords:** Inborn errors of metabolism, Lysosomal storage diseases, Sphingolipids, Sphingolipidoses, Gaucher disease, Fabry disease, Rheumatic diseases, Rare diseases

## Abstract

Gaucher and Fabry diseases are lysosomal storage disorders in which deficient enzyme activity leads to pathological accumulation of sphingolipids. These diseases have a broad phenotypic presentation. Musculoskeletal symptoms and pain complaints are frequently reported by patients. Thus, rheumatologists can be contacted by these patients, contributing to the correct diagnosis, earlier indication of appropriate treatment and improvement of their prognosis. This review describes important concepts about Gaucher and Fabry diseases that rheumatologists should understand to improve patients’ quality of life and change the natural history of these diseases.

## Introduction

Gaucher disease (GD) and Fabry disease (FD) are inherited metabolic disorders (IMDs) in which deficient activity of an enzyme leads to pathological accumulation of lysosomal substrates. Thus, these conditions are classified as lysosomal storage diseases and belong to the group of sphingolipidoses, since the metabolic defect is related to the degradation of sphingolipids. Both diseases present wide phenotypic variation, ranging from mild, oligosymptomatic cases to severe and life-threatening conditions [1, 2].

Patients with GD or FD may seek a rheumatologist due to complaints of diffuse or localized pain, in addition to systemic manifestations. Therefore, rheumatologists should be prepared to consider the main characteristics of GD and FD in the differential diagnosis of rheumatic conditions, contributing to the early diagnosis and treatment of these sphingolipidoses [3]. This article presents an overview of GD and FD in the context of inherited metabolic disorders and lysosomal storage diseases, with an emphasis on relevant aspects to the clinical practice of rheumatologists.

## Inherited metabolic disorders

IMDs are caused, in most cases, by the deficiency of an enzyme or its cofactor, which leads to dysfunction of one or more metabolic pathways. Most IMDs result from a loss-of-function pathogenic variant in a single gene. However, some IMDs are associated with gain-of-function variants or structural genetic changes [4, 5]. Although these diseases are generally rare from an individual perspective, collectively their incidence has been reported to be 1:800 to 1:1500 live births [6, 7].

IMDs have a broad clinical spectrum. Most symptomatic patients are diagnosed during childhood, but some, especially those with milder forms, may be diagnosed in adulthood. Some IMDs are managed with specific diets, as in phenylketonuria. Others have had their management revolutionized with therapeutic modalities such as enzyme replacement therapy (ERT), chaperone therapy, substrate reduction therapy (SRT), and bone marrow transplantation. Gene therapy and genome editing seem promising in the field [5, 8]. One of the main challenges is diagnosing these diseases in a timely manner to achieve the best results with early initiation of therapy [8, 9].

The International Classification of Inherited Metabolic Disorders incorporates 1450 conditions, divided into 24 categories: (1–13) disorders of intermediary metabolism; (14–15) disorders of lipid metabolism and transport; (16–17) disorders of heterocyclic compounds; (18–20) disorders that affect the metabolism of complex macromolecules and organelles; (21–22) disorders of the metabolism of cofactors and minerals; (23–24) disorders of metabolic cell signaling [4].

Category number 20 (disorders of complex molecules degradation) includes lysosomal storage diseases (LSDs) caused by enzyme deficiencies, which are related to defects in the degradation of sphingolipids, glycosaminoglycans, or glycoproteins [4].

## Lysosomal storage disorders

Lysosomes are intracellular organelles responsible for degrading and recycling complex carbohydrates, proteins, lipids, and nucleic acids. Deficiency of lysosomal enzymes or other lysosomal proteins may lead to the accumulation of nondegraded or partially degraded macromolecules, resulting in progressive cellular dysfunction [10]. LSDs represent a heterogeneous group of multisystem conditions, ranging from rapidly fatal diseases to attenuated but highly morbid forms in adulthood. Most of them are inherited in an autosomal recessive manner; some exceptions are inherited with an X-linked pattern [10, 11].

A study using retrospective data of IMDs diagnosed in the United Kingdom (West Midlands) from 1999 to 2003 estimated a birth prevalence of approximately 1 in 5000 for LSDs [7]. Currently, more than 50 monogenic disorders of lysosomal catabolism have been recognized. The majority of them are caused by enzyme deficiencies and can be subclassified according to the nature of the accumulated substrate (e.g., sphingolipidoses, mucopolysaccharidoses and glycoproteinoses). Other types of lysosomal conditions include disorders of post-translational modification of lysosomal hydrolases; disorders of integral membrane proteins, disorders of lysosome-related organelle biogenesis, and disorders of lipofuscin production [11].

## Sphingolipidoses

Sphingolipids are bioactive signaling lipid molecules that can be found in plasma membranes. They participate in a variety of cellular functions and have been linked not only to LSDs, but also to inflammatory disorders, cancer, and metabolic and neurodegenerative diseases [12].

Structurally, sphingolipids are composed of a sphingoid base backbone upon which additional functional groups can be added. The most common sphingoid base is sphingosine, a long-chain amino alcohol. Based on their additional groups and complexity, sphingolipids are classified into sphingomyelins, cerebrosides, gangliosides, globosides, among others [8, 12, 13].

Complex sphingolipids are removed from plasma membranes and catabolized mainly through the lysosomal digestion system. Defects in the hydrolysis of sphingolipids due to deficiency of enzymes or other proteins needed for lysosomal degradation lead to the development of sphingolipidoses [11–14]. Table 1 shows the diseases that belong to the group of sphingolipidoses, the corresponding gene, the enzyme/protein deficiency, the accumulated substrate, and the main clinical features.

**Table 1:** Sphingolipidoses

Disease	Gene	Enzyme/protein deficiency	Accumulated substrate	Main clinical features
Gaucher disease	*GBA*	β-Glucocerebrosidase	Glucocerebroside and glucosylsphingosine	Type I (non-neuronopathic): hepatosplenomegaly, thrombocytopenia, bone complications, pulmonary disease. Type II: early onset and short life expectancy, visceral and bone marrow involvement, spasticity, severe neurological manifestations. Type III: visceral and bone marrow involvement, less severe neurological involvement than type II.
Fabry disease	*GLA*	α-Galactosidase A	Globotriaosylceramide	Males: cornea verticillata, acroparesthesia, angiokeratomas, gastrointestinal symptoms, hypohidrosis, progressive organ failure (cardiomyopathy, kidney and cerebrovascular disease). Females range from no symptoms to severe manifestations as males.
Farber disease	*ASAH1*	Acid ceramidase	Ceramide	Type I: early onset and premature death, organomegaly, joint contractures, voice hoarseness, neurological manifestations. Type II: intermediate. Type III: mild. Type IV: neonatal-visceral. Type V: neurological-progressive. Type VI: combined Farber and Sandhoff diseases.
GM1 gangliosidosis	*GLB1*	β-Galactosidase	GM1 ganglioside, keratan sulfate and oligosaccharides	Type I: premature death, severe neurological manifestations, organonomegaly, skeletal abnormalities, blindness and deafness. Type II: developmental delay, dementia, cerebellar signs, late loss of vision. Type III: dysarthia, gait disturbances, dystonia, cardiomyopathy.
GM2 gangliosidosis (Tay–Sachs disease)	*HEXA*	β-Hexosaminidase α subunit	GM2 ganglioside, glycosphingolipids and oligosaccharides	Weakness, bone abnormalities, neurological manifestations, reduction of consciousness, vision, and hearing.
GM2 gangliosidosis (Sandhoff disease)	*HEXB*	β-Hexosaminidase β subunit	GM2 ganglioside, GA2 glycolipid and oligosaccharides	Neurological manifestations, less bone involvement than Tay-Sachs disease.
GM2 gangliosidosis (GM2 activator deficiency)	*GM2A*	GM2 ganglioside activator	GM2 ganglioside and glycosphingolipids	Weakness, seizures, loss of vision and hearing, intellectual disability, paralysis.
Globoid cell leukodystrophy (Krabbe disease)	*GALC*	Galactosylceramidase	Galactocerebroside and psychosine	Psychomotor dysfunction, seizures, spasticity, cognitive decline.
Metachromatic leukodystrophy	*ARSA* and *PSAP*	Arylsulfatase A and prosaposin	Sulfatides	Unsteady gait, mental regression, seizures, unsteady gait, incontinence, blindness, loss of motor function. Adult form: variable progression.
Niemann–Pick disease types A and B	*SMPD1*	Sphingomyelin phosphodiesterase	Sphingomyelin	Type A: early onset and premature death, lymphadenopathy, organonomegaly, weakness, dysphagia, severe psychomotor dysfunction. Type B: slowly progressive symptoms, no neurodegeneration, variable visceral involvement, organomegaly, liver dysfunction, and lung disease.

Adapted from reference [8]

GD and FD are among the most common LSDs. As previously mentioned, these sphingolipidoses are relevant conditions for the clinical practice of rheumatologists, as they can present with pain syndromes and musculoskeletal symptoms, mimicking rheumatic diseases [5, 10, 15].

## Gaucher disease

### General aspects

**Fig. 1:**
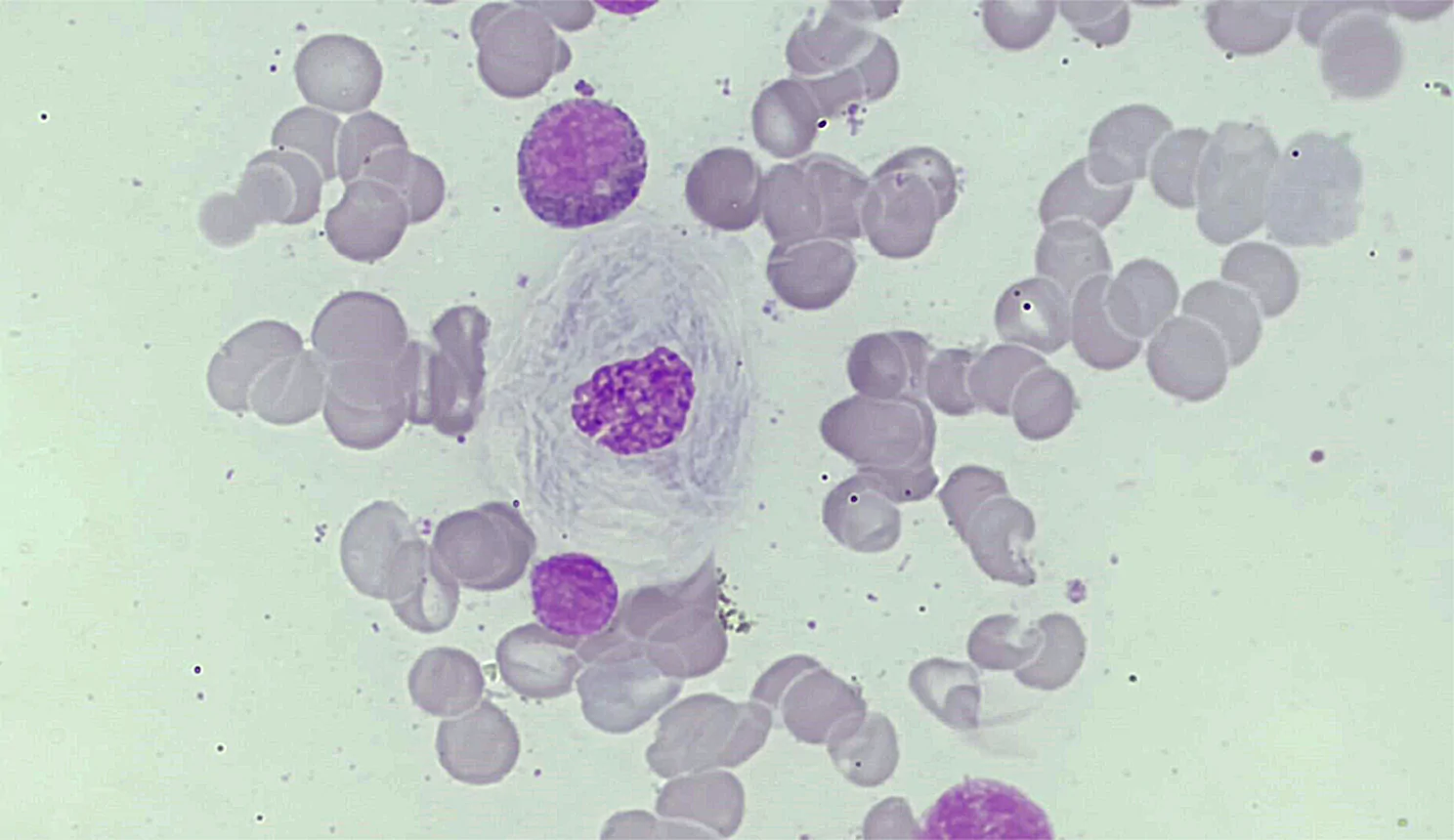
Bone marrow aspirate smear [Leishman stain, 1000 ×]. Note the Gaucher cell, a sphingolipid-laden macrophage with abundant cytoplasm that looks like “wrinkled paper”. Courtesy of Dr. Felipe Melo Nogueira

GD is the most common sphingolipidosis with an estimated birth incidence of 0.39 to 5.8 per 100,000 in the general population [11, 16]. It is an autosomal recessive disease caused by the deficiency of lysosomal β-glucocerebrosidase (β-GCase), also known as glucosylceramidase, due to pathogenic variants in the *GBA* gene. This enzyme deficiency leads to the accumulation of glucocerebroside (glucosylceramide) and related compounds, especially in the macrophage-monocyte system. Sphingolipid-laden macrophages with a “wrinkled paper” (or “crumpled silk”) appearance in the cytoplasm are called Gaucher cells and are typically found in the bone marrow [17] (Fig. 1).

GD is classified into three main subtypes: type 1 (non-neuronopathic form), type 2 (acute neuronopathic form), and type 3 (subacute/chronic neuronopathic form) [16]. GD type 1 (GD1) accounts for more than 90% of all GD cases; it has a much higher prevalence in Ashkenazi Jews (118 per 100,000) than in non-Jewish populations (1–2 per 100,000) [16, 18, 19]. The age of onset is highly variable, and patients can be diagnosed in childhood, adolescence, or adulthood [18, 20]. GD1 has a broad clinical spectrum, ranging from mild symptoms to severe manifestations, including hepatosplenomegaly, hematological abnormalities, bone complications, pulmonary disease, and increased risk of malignancies. Although GD1 is called non-neuronopathic, patients are at increased risk of developing Parkinson-like syndromes [18, 19].

GD type 2 (GD2) and GD type 3 (GD3) are dominated by neurologic manifestations, although extraneurological disease can also occur [16]. Patients with GD2 and GD3 present inflammation and neuronal loss in the central nervous system [21]; the clinical picture is frequently marked by impaired psychomotor development, abnormal saccadic eye movements, spasticity, and seizures [16]. GD2 is the most severe form, with an early onset, rapidly progressive course, and very short life expectancy (most of these patients die before 3 years of age) [22].

### Relevance for rheumatologists

Musculoskeletal manifestations are highly prevalent and associated with significant morbidity and reduced quality of life in patients with GD [23]. Skeletal disease includes bone marrow infiltration by Gaucher cells, microvascular bone occlusion, bone infarction, osteonecrosis, cortical thinning, abnormal remodeling with osteolytic lesions, and loss of bone mineral density (BMD). As a result, patients may present with diffuse or localized bone pain, fragility fractures, joint collapse with secondary osteoarthritis, axial and appendicular deformities, and a high degree of disability [23, 24].

**Fig. 2:**
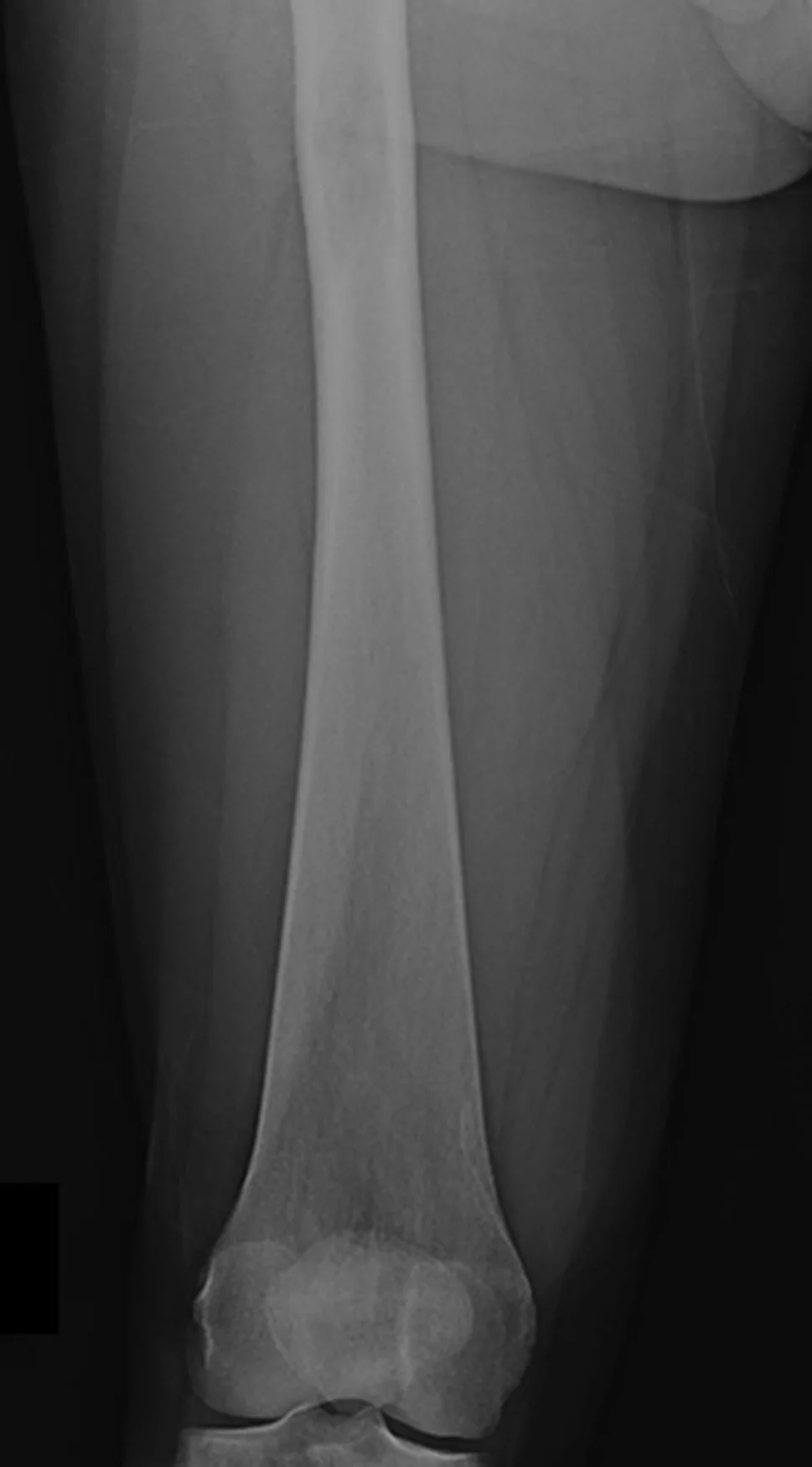
Plain radiograph of the right distal femur of a patient with Gaucher disease under enzyme replacement therapy. Note the Erlenmeyer flask deformity with enlargement of the metaphyseal region. Courtesy of Dr. Diogo Souza Domiciano

A report from the International Collaborative Gaucher Group of 1698 patients in the Gaucher Registry (94% with GD1) showed that 63% of patients experienced bone pain and more than 90% had radiographic evidence of bone involvement, independently of bone symptoms [18]. The most common radiological finding was the Erlenmeyer flask deformity, which results from defective bone remodeling and enlargement of metaphyseal region of long bones, especially the distal femur [18, 23, 24] (Fig. 2). Regarding fragility fractures, a study with consecutive adult patients from 3 referral centers in the United Kingdom (96 with GD1 and 4 with GD3; median age 49 years) found this outcome in 28% of the sample [25]. Another study with a cohort of French GD1 patients (mean age 45 years) reported an overall prevalence of 18% of nonvertebral fractures and 15% of vertebral fractures [26].

The pathogenesis of GD-related osteoporosis is not completely understood. The disease affects the bone marrow and mineralized bone tissue. There is evidence that increased osteoclast activation and high bone resorption are influenced by cytokines produced by activated macrophages [27, 28]. The assessment of BMD through dual-energy X-ray absorptiometry (DXA) should take into account bone changes in GD, mainly osteonecrosis of femoral head and vertebral bodies, and also fractured vertebrae, which overestimate BMD and should be excluded from the analysis [23].

It is also important to always be aware of the increased risk of malignancies in GD patients, especially multiple myeloma (approximately nine times higher than expected for the general population), which are frequently associated with osteolytic lesions and pathological fractures [29].

### Diagnosis

The diagnosis of GD is confirmed by the demonstration of low β-GCase activity (<15% of mean normal activity) in peripheral leukocytes or extracts of cultured skin fibroblasts [30]. The diagnosis can also be confirmed by the identification of biallelic pathogenic variants in the *GBA* gene. According to the guidelines developed by the International Working Group of Gaucher Disease, the gold standard for GD diagnosis is the demonstration of deficient β-GCase activity, supported by genetic testing to corroborate the diagnosis and to provide genetic family counseling [31].

Enzymatic activity levels are not suitable for identifying heterozygous carriers of pathogenic variants in the *GBA* gene or patients with atypical GD due to deficiency of saposin C, the β-GCase activator encoded by the *PSAP* gene [31, 32]. Genetic testing is the method of choice for detecting heterozygotes for pathogenic variants among family members of patients with GD. When genetic testing is performed as the primary test (i.e., before β-GCase activity), the physician should be aware of the limitations of the method, since some genetic variants may lead to misinterpretation or be missed in DNA sequencing technologies. Furthermore, finding variants of uncertain significance is not uncommon [31, 33].

### Treatment

Treatment for GD is well established for symptomatic children or adults with GD1. There are no highly effective treatments for the neurological manifestations of the neuronopathic forms (GD2 and GD3) [30, 34, 35].

ERT with recombinant glucocerebrosidases (imiglucerase, velaglucerase alfa, or taliglucerase alfa) in GD1 aims to increase hemoglobin levels and platelet counts, eliminate transfusional dependence; reduce splenic and liver volume; reduce bone pain due to bone marrow infiltration; decrease the risk of new episodes of osteonecrosis and fragility fractures; and prevent impaired growth with early treatment in children [30, 35]. SRT in GD (eliglustat and miglustat) is based on the inhibition of glucosylceramide synthase, which participates in the biosynthesis of glycosphingolipids [30, 35]. ERT has reduced the indications for splenectomy in GD, currently reserved for rare circumstances such as persistent and severe cytopenia with high risk of bleeding [30, 35].

Biphosphonates are considered adjunctive therapies for GD patients with markedly reduced BMD, although there are no robust evidence-based guidelines on how to manage antiresorptive agents specifically in this population [23, 30].

## Fabry disease

### General aspects

FD is the second most common sphingolipidosis after GD. It is a pan-ethnic X-linked disease with an estimated birth incidence of 1 per 117,000 in the general population, and 1 per 40,000 male live births [8, 36, 37]. FD is caused by deficient or absent lysosomal α-galactosidase A (α-GAL) activity. This enzyme is encoded by the *GLA* gene located on the long arm of the X chromosome. More than 1000 *GLA* pathogenic or likely pathogenic variants have been described to date [8, 38].

α-GAL catalyzes the removal of terminal galactose groups from substrates such as globotriaosylceramide (Gb3) and glycoproteins [39]. Thus, the main metabolic consequence of α-GAL deficiency is the accumulation of Gb3 and its deacylated form globotriaosylsphingosine (lyso-Gb3) in multiple cells, especially in the vascular endothelium, vascular smooth muscle cells, cardiomyocytes, podocytes, autonomic ganglia, and conduction fibers [38–41].

**Fig. 3:**
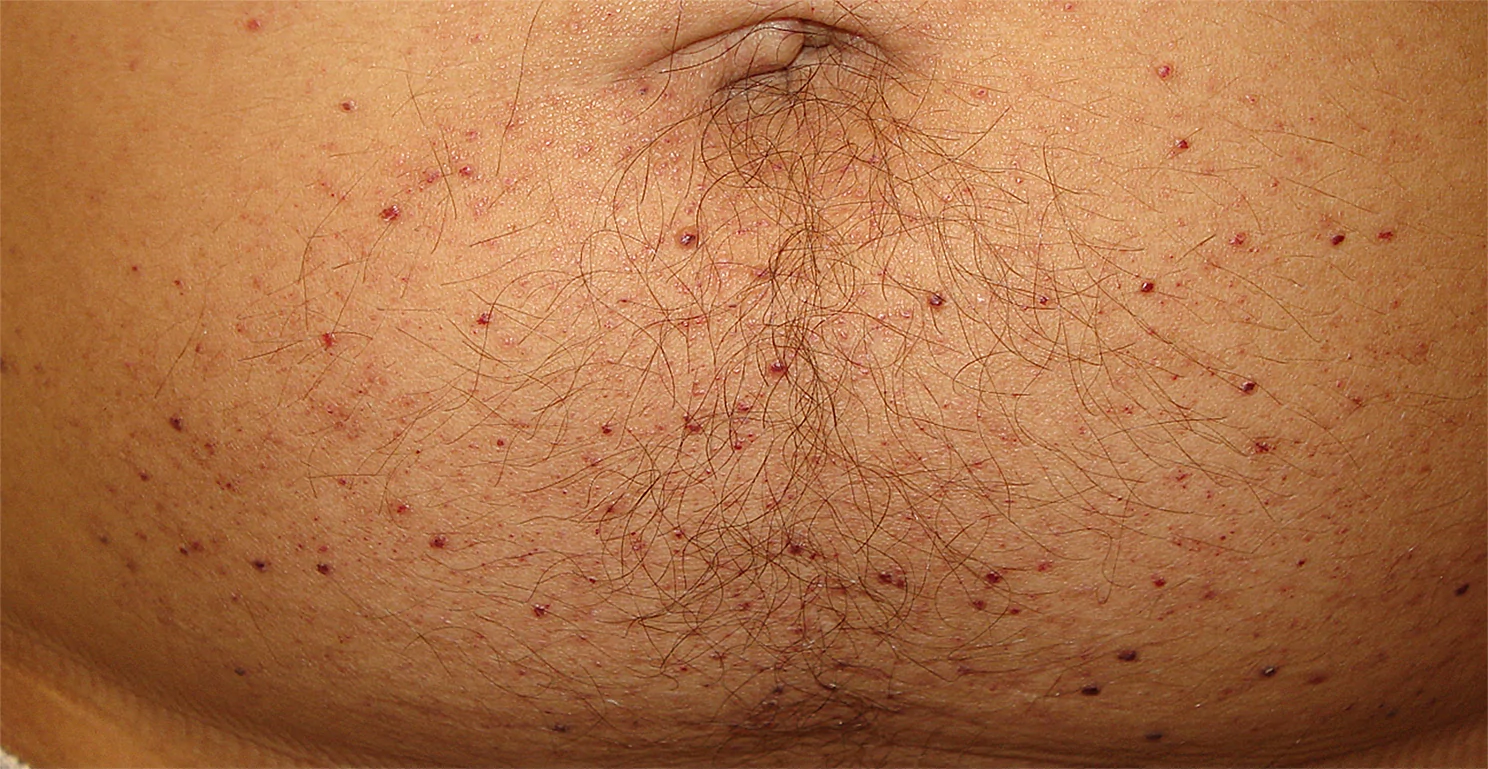
Multiple reddish-purple angiokeratomas in the lower abdomen of a patient with Fabry disease. Courtesy of Dr. Nilton Salles Rosa Neto

Manifestations of classic FD begin in childhood or adolescence. Classic FD is observed in hemizygous males, who have minimal or no enzymatic activity of α-GAL [42, 43]. Patients may present with a variety of manifestations, including neuropathic pain in the extremities (acroparesthesias); hypohidrosis; heat, cold, and exercise intolerance; gastrointestinal symptoms, such as recurrent abdominal pain, nausea, intermittent diarrhea, or constipation; unexplained fever; angiokeratomas, characterized as nonblanching red to bluish-black papules, most commonly on the trunk, groin, and periumbilical areas (Fig. 3); and corneal opacities (cornea verticillata) seen on slit lamp examination [42–44]. In adulthood, progressive organ failure becomes more evident with the following involvements: cardiac (left ventricular hypertrophy, arrhythmias, coronary artery disease, and heart failure), cerebrovascular (transient ischemic attacks and strokes), and renal (proteinuria and deterioration of kidney function) [42, 43].

Female patients with FD are most frequently heterozygous for pathogenic variants in the *GLA* gene. They have a broad phenotypic presentation that ranges from no manifestation to a severe phenotype similar to classic FD, usually observed in hemizygous males [43, 45]. One explanation for this wide phenotypic variation in females is the skewed inactivation of one X chromosome in each cell during embryogenesis. Thus, clinical manifestations depend on the proportion of cells with the X chromosome that express the mutated *GLA* gene [46]. There are also male and female patients with variants of late-onset FD, who tend to have disease predominantly affecting the heart or the kidney [47–49].

### Relevance for rheumatologists

FD is a relatively rare disease, with a wide variety of nonspecific manifestations. The lack of recognition of FD manifestations by the medical community may explain, at least in part, the significant delay in diagnosis, reported in the literature as a median time of 16 to 18 years [50–52]. Rheumatology may be one of the specialties sought by patients with FD during their journey to diagnosis. In fact, many FD manifestations may mimic rheumatic conditions, and it is not rare for these patients to be misdiagnosed and incorrectly treated [50–52].

A retrospective study that analyzed the medical records of 107 adult patients with FD found that 26.2% of them received at least one incorrect diagnosis of rheumatic disease. The authors argued that arthralgia, unexplained fever, Raynaud’s phenomenon, and episodes of elevated inflammatory markers were possible reasons for the incorrect diagnosis of a rheumatic condition [51]. Another study conducted with 37 Brazilian patients with definite FD reported that diagnostic errors occurred in 64.8% of the sample, and the majority of the incorrect diagnoses were musculoskeletal/rheumatic conditions, such as rheumatic fever, “unspecified rheumatism”, growing pains, and fibromyalgia [52].

In children and adolescents, rheumatologists should be alert to acroparesthesias, hypohidrosis, angiokeratomas, intolerance to exercise, heat or cold, and fever attacks with painful extremities without synovial inflammation as clues for early diagnosis of FD. A detailed family history is another extremely helpful point for diagnosing FD [53–56].

FD should be included in the differential diagnosis of recurrent fever of unknown origin, especially when associated with one or more of the characteristics mentioned above [57, 58]. In rheumatology practice, at first glance, fibromyalgia can also be confused with FD due to a history of multiple symptoms, including chronic pain and gastrointestinal complaints, which could be interpreted as irritable bowel syndrome in the context of central sensitization. However, a thorough history and physical examination may reveal atypical features not explained by fibromyalgia alone, although fibromyalgia syndrome may be present as a comorbidity in patients with FD [52, 59, 60].

### Diagnosis

The diagnosis of FD is preferably made by the determination of α-GAL activity and/or genetic testing. In males with compatible FD phenotype, a very low leukocyte α-GAL activity (<5% of mean normal activity) is sufficient to establish the diagnosis. In females, genetic testing with detection of disease-causing variant in the *GLA* gene is required for the diagnosis of FD, since heterozygous females have variable α-GAL activity levels, ranging from normal to very low [42, 61, 62].

Mutational analysis of the *GLA* gene is useful regardless of sex, because it facilitates genetic counseling and allows identification of the amenability of gene variants to chaperone therapy [61, 63]. A small number of pathogenic variants, particularly in the case of duplications/deletions and deep intronic variants, may escape detection by routine analysis and can only be identified by a more sophisticated assessment of the *GLA* gene [61].

### Treatment

Treatment for FD should be considered for all male patients, symptomatic or asymptomatic, with a pathogenic variant known to be associated with classical disease, regardless of the age of presentation. For females, treatment should be considered for symptomatic patients and/or those with evidence of major organ involvement. Asymptomatic females without laboratory, histological, or imaging evidence of renal, cardiac, or central nervous system involvement may not receive specific treatment but should be monitored regularly. Treatment should also be considered for male and female patients with later-onset variants and single-organ disease, provided that the abnormalities are attributable to FD [62].

FD-specific treatment consists of ERT, which involves biweekly intravenous infusions of recombinant α-GAL (agalsidase alfa, agalsidase beta, or pegunigalsidase alfa), or oral chaperone therapy (migalastat hydrochloride) administered every other day [64–66]. Treatment with ERT is recommended for both adults and children, but the age at which it is authorized varies according to the product and the approval specific to each country. Chaperone therapy stabilizes some misfolded forms of α-GAL, which facilitates their trafficking to lysosomes, prolongs their half-life and enhances their catalytic activity [66, 67]. Treatment with migalastat is intended for patients who are 12 years of age or older, have amenable GLA gene variants, and have an estimated glomerular filtration rate (eGFR) greater than 30 mL/min/1.73 m^2^. Amenability refers to the ability of the α-GAL encoded by amenable variants to respond to chaperone therapy in vitro with an increase in its activity. However, this does not necessarily imply a response in vivo, which should be monitored at the discretion of the attending physician [66, 67].

The therapeutic goals of specific treatments for FD are to improve quality of life and exercise tolerance; prevent the development or stabilize the progression of left ventricular hypertrophy, myocardial fibrosis, arrhythmias, and heart failure; reduce proteinuria and prevent the development or stabilize the progression of eGFR decline; decrease the risk of ischemic cerebral events; reduce the intensity of neuropathic pain and/or frequency of pain crises; and reduce gastrointestinal symptoms [42, 68].

Nonspecific treatments also play an important role in the management of FD and can include, but are not limited to, optimal blood pressure control; insertion of an implantable cardioverter defibrillator in patients with sustained ventricular arrhythmia or other high-risk situations; pacemaker implantation in patients with atrioventricular block; oral anticoagulation for atrial fibrillation; renal replacement therapy or kidney transplantation for end-stage kidney disease; and symptomatic treatment of chronic neuropathic pain [42, 62, 68, 69].

## Conclusion

GD and FD are complex and heterogeneous diseases that are often either misdiagnosed or diagnosed late. Rheumatologists may encounter these patients in clinical practice due to their musculoskeletal features and pain complaints. Therefore, rheumatologists should familiarize themselves with the main clinical characteristics of these diseases so as not to miss the opportunity for timely recognition and early treatment, which can result in a better prognosis.

## Data Availability

Not applicable.

## List of abbreviations

GD: Gaucher disease
FD: Fabry disease
IMDs: Inherited metabolic disorders
ERT: Enzyme replacement therapy
SRT: Substrate reduction therapy
LSDs: Lysosomal storage disorders
β-GCase: Β-glucocerebrosidase
GD1: Gaucher disease type 1
GD2: Gaucher disease type 2
GD3: Gaucher disease type 3
BMD: Bone mineral density
DXA: Dual-energy X-ray absorptiometry
α-GAL: α-galactosidase A
Gb3: Globotriaosylceramide
lyso-Gb3: Globotriaosylsphingosine
